# Mental health trajectories and Peer Refugee Helper engagement, among Afghan, Iranian and Syrian refugees and asylum seekers in Greece – CORRIGENDUM

**DOI:** 10.1017/gmh.2026.10213

**Published:** 2026-06-01

**Authors:** Michalis Lavdas, Gro Mjeldheim Sandal, Marit Sijbrandij, Trynke Hoekstra, Tormod Bøe

The authors regret the inclusion of an error in the above article.

On page 3, Table 1, data values were incorrect as follows:

**GAD–7 score**

Original value: 15.5 (10.1)

Correct value: 8.87 (5.74)

**PHQ–9 score**

Original value: 19.9 (12.5)

Correct value: 11.07 (6.97)

All statistical analyses, figures, and interpretations reported in the manuscript were conducted using the correct values. Therefore, the error does not affect the results or conclusions of the study and is limited to the descriptive tables.

These values have been corrected in the article.

These values have also been updated in the supplementary material, along with the p-value^2^ as follows:

**GAD–7 score**

Original p-value^2^: >0.9

Correct p-value^2^: 0.9

**PHQ–9 score**

Original p-value^2^: 0.5

Correct p-value^2^: 0.4

and the T2 values as follows:

**GAD–7 score**

Original value: 16.07 (10.44)

Correct value: 9.05 (5.98)

**PHQ–9 score**

Original value: 17.93 (12.16)

Correct value: 9.93 (6.74)
