# Supplementary material for: Mental health trajectories and Peer Refugee Helper engagement, among Afghan, Iranian and Syrian refugees and asylum seekers in Greece
Source: Glob Ment Health (Camb). 2025 Oct 13;12:e115. doi: 10.1017/gmh.2025.10068 (PMC12571684; doi:10.1017/gmh.2025.10068)
Supplement: Lavdas et al. supplementary material [file S205442512510068Xsup001.zip › S205442512510068Xsup001.docx]

**Supplementary Material**

I. Applied mixed models and latent class mixed models for Gaussian longitudinal outcomes (*hlme* *function*), introducing in two steps random intercept and slope, with quadratic time structure. Indicators for 1-5 Models for GAD-7 and PHQ-9

A. GAD-7 LGMM with random intercept

| G | loglik | conv | npm | AIC | BIC | SABIC | entropy | %class1 | %class2 | %class3 | %class4 | %class5 |
| --- | --- | --- | --- | --- | --- | --- | --- | --- | --- | --- | --- | --- |
| 1 | -899.1275 | 1 | 5 | 1,808.255 | 1,824.079 | 1,808.245 | 1.0000000 | 100.000000 |  |  |  |  |
| 2 | -897.1704 | 1 | 9 | 1,812.341 | 1,840.824 | 1,812.324 | 0.4627574 | 26.857143 | 73.14286 |  |  |  |
| 3 | -892.2872 | 1 | 13 | 1,810.574 | 1,851.717 | 1,810.550 | 0.7148712 | 54.857143 | 24.00000 | 21.142857 |  |  |
| 4 | -887.1807 | 1 | 17 | 1,808.361 | 1,862.163 | 1,808.329 | 0.7299195 | 52.571429 | 22.85714 | 6.285714 | 18.28571 |  |
| 5 | -877.9612 | 1 | 21 | 1,797.922 | 1,864.383 | 1,797.882 | 0.7404666 | 7.428571 | 16.00000 | 45.714286 | 21.71429 | 9.142857 |

[insert supplementary figure A]

B. GAD-7 LGMM with random intercept and slope

| G | loglik | conv | npm | AIC | BIC | SABIC | entropy | %class1 | %class2 | %class3 | %class4 | %class5 |
| --- | --- | --- | --- | --- | --- | --- | --- | --- | --- | --- | --- | --- |
| 1 | -898.5647 | 1 | 10 | 1,817.129 | 1,848.777 | 1,817.110 | 1.0000000 | 100.00000 |  |  |  |  |
| 2 | -1,000,000,000.0000 | 4 | 14 | 2,000,000,028.000 | 2,000,000,072.307 | 2,000,000,027.973 | 1.0000000 | 0.00000 | 0.00000 |  |  |  |
| **3** | **-882.3222** | **1** | **18** | **1,800.644** | **1,857.611** | **1,800.610** | **0.8023711** | **22.85714** | **48.00000** | **29.14286** |  |  |
| 4 | -884.3883 | 1 | 22 | 1,812.777 | 1,882.402 | 1,812.735 | 0.6437702 | 12.57143 | 45.14286 | 16.57143 | 25.71429 |  |
| 5 | -878.7238 | 1 | 26 | 1,809.448 | 1,891.732 | 1,809.398 | 0.6448836 | 33.71429 | 16.57143 | 20.57143 | 11.42857 | 17.71429 |

[insert supplementary figure B]

C. PHQ-9 LGMM with random intercept

| G | loglik | conv | npm | AIC | BIC | SABIC | entropy | %class1 | %class2 | %class3 | %class4 | %class5 |
| --- | --- | --- | --- | --- | --- | --- | --- | --- | --- | --- | --- | --- |
| 1 | -959.7777 | 1 | 5 | 1,929.555 | 1,945.351 | 1,929.518 | 1.0000000 | 100.000000 |  |  |  |  |
| 2 | -957.0093 | 1 | 9 | 1,932.019 | 1,960.450 | 1,931.951 | 0.9283655 | 1.149425 | 98.85057 |  |  |  |
| 3 | -954.2521 | 1 | 13 | 1,934.504 | 1,975.572 | 1,934.406 | 0.6146636 | 51.724138 | 47.12644 | 1.149425 |  |  |
| 4 | -943.6438 | 1 | 17 | 1,921.288 | 1,974.991 | 1,921.159 | 0.6376128 | 5.172414 | 51.72414 | 37.931034 | 5.172414 |  |
| 5 | -947.7358 | 1 | 21 | 1,937.472 | 2,003.812 | 1,937.313 | 0.6358733 | 8.045977 | 36.20690 | 21.839080 | 11.494253 | 22.41379 |

[insert supplementary figure C]

D. PHQ-9 LGMM with random intercept and slope

| G | loglik | conv | npm | AIC | BIC | SABIC | entropy | %class1 | %class2 | %class3 | %class4 | %class5 |
| --- | --- | --- | --- | --- | --- | --- | --- | --- | --- | --- | --- | --- |
| 1 | -959.3994 | 1 | 10 | 1,938.799 | 1,970.389 | 1,938.723 | 1.0000000 | 100.00000 |  |  |  |  |
| **2** | **-952.8493** | **1** | **14** | **1,933.699** | **1,977.925** | **1,933.593** | **0.6212593** | **37.93103** | **62.06897** |  |  |  |
| 3 | -946.4985 | 1 | 18 | 1,928.997 | 1,985.860 | 1,928.861 | 0.6324579 | 40.80460 | 52.29885 | 6.896552 |  |  |
| 4 | -939.2345 | 1 | 22 | 1,922.469 | 1,991.968 | 1,922.303 | 0.7066353 | 37.93103 | 52.29885 | 2.298851 | 7.471264 |  |
| 5 | -937.6926 | 1 | 26 | 1,927.385 | 2,009.521 | 1,927.188 | 0.6369456 | 31.60920 | 27.58621 | 2.298851 | 9.195402 | 29.31034 |

[insert supplementary figure D]

II. Table Presenting Comparison between baselines T1 and T2

| Descriptive Statistics | **N** | **T1** N = 153*^1^* | **T2** N = 23*^1^* | **p-value***^2^* |
| --- | --- | --- | --- | --- |
| **Age** | 176 | 34.86 (9.14) | 36.61 (11.92) | 0.8 |
| **Gender** | 176 |  |  | 0.8 |
| Male |  | 68 (44.44%) | 9 (39.13%) |  |
| Female |  | 80 (52.29%) | 13 (56.52%) |  |
| Non-binary/Not disclosed |  | 5 (3.27%) | 1 (4.35%) |  |
| **Country of Origin** | 154 |  |  | 0.6 |
| Afghanistan |  | 39 (25.83%) | 2 (66.67%) |  |
| Iran |  | 55 (36.42%) | 1 (33.33%) |  |
| Other |  | 24 (15.89%) | 0 (0%) |  |
| Syrian Arab Republic |  | 33 (21.85%) | 0 (0%) |  |
| Missing |  | 2 | 20 |  |
| **Years in School** | 175 | 10.66 (5.80) | 8.78 (4.51) | 0.08 |
| Missing |  | 1 | 0 |  |
| ***Years in Greece** | 174 | 7.17 (6.27) | 8.22 (2.95) | 0.02 |
| Missing |  | 2 | 0 |  |
| **Children** | 146 |  |  | 0.4 |
| No children |  | 32 (25.60%) | 3 (14.29%) |  |
| 1-2 children |  | 63 (50.40%) | 15 (71.43%) |  |
| 3-4 children |  | 23 (18.40%) | 2 (9.52%) |  |
| More than 4 |  | 7 (5.6%) | 1 (4.76%) |  |
| Missing |  | 28 | 2 |  |
| **Legal status** | 175 |  |  | 0.1 |
| Refugee status |  | 88 (57.89%) | 8 (34.78%) |  |
| Asylum seeker |  | 45 (29.61%) | 10 (43.48%) |  |
| Other |  | 19 (12.50%) | 5 (21.74%) |  |
| Missing |  | 1 | 0 |  |
| **Occupation** | 170 |  |  | 0.6 |
| Other |  | 42 (28.38%) | 9 (40.91%) |  |
| Paid Work |  | 39 (26.35%) | 4 (18.18%) |  |
| Unemployed |  | 52 (35.14%) | 8 (36.36%) |  |
| Volunteer |  | 15 (10.14%) | 1 (4.55%) |  |
| Missing |  | 5 | 1 |  |
| **Number of Traumatic Events** | 175 | 3.42 (2.54) | 3.09 (2.78) | 0.5 |
| Missing |  | 0 | 1 |  |
| **PRH Status** | 170 |  |  | 0.11 |
| Paid PRH |  | 29 (19.59%) | 1 (4.55%) |  |
| Paid Non-Helper |  | 10 (6.76%) | 3 (13.64%) |  |
| Unpaid PRH |  | 48 (32.43%) | 5 (22.73%) |  |
| Unpaid Non-Helper |  | 61 (41.22%) | 13 (59.09%) |  |
| Missing |  | 5 | 1 |  |
| **Living situation** | 175 |  |  | 0.14 |
| Living without partner |  | 78 (51.32%) | 8 (34.78%) |  |
| Living with partner |  | 74 (48.68%) | 15 (65.22%) |  |
| Missing |  | 1 | 0 |  |
| ***Accommodation** | 136 |  |  | 0.04 |
| Refugee Camp |  | 14 (12.39%) | 5 (21.74%) |  |
| Urban Area |  | 83 (73.45%) | 11 (47.83%) |  |
| Other |  | 16 (14.16%) | 7 (30.43%) |  |
| Missing |  | 40 | 0 |  |
| **GAD-7 Score** | 172 | 15.54 (10.05) | 16.07 (10.44) | >0.9 |
| Missing |  | 3 | 1 |  |
| **PHQ-9 Score** | 172 | 19.94 (12.54) | 17.93 (12.16) | 0.5 |
| Missing |  | 3 | 1 |  |
| **SOC Score** | 171 | 49.62 (12.94) | 48.69 (15.59) | >0.9 |
| Missing |  | 4 | 1 |  |
| **SPS Score** | 168 | 65.86 (12.82) | 69.45 (13.18) | 0.3 |
| Missing |  | 7 | 1 |  |
| ***PACT score** | 167 | 0.60(0.59) | 1.09(1.02) | 0.02 |
|  |  | 7 | 2 |  |
| *^1^* Mean (SD); n (%) | | | | |
| *^2^* Wilcoxon rank sum test; Fisher’s exact test; Pearson’s Chi-squared test | | | | |
| *Notes.* PRH = Peer Refugee Helper; GAD-7 = Generalized Anxiety Disorder scale; PHQ-9 = Patient Health Questionnaire; SOC-13 = Sense of Coherence; SPS = Social Provisions Scale; PACT = Perceived Ability to Cope With Trauma. | | | | |

III. Visualized Odds Ratios after adjusting for potential confounders for belonging to the low depression trajectory

[insert supplementary figure E]

IV. Visualized Odds Ratios after adjusting for potential confounders for belonging to the low anxiety and moderate anxiety trajectory

***GAD-7 Model Odds Ratios for Low Anxiety Trajectory***

[insert supplementary figure F]

***GAD-7 Model Odds Rations for Moderate Anxiety Trajectory***

[insert supplementary figure G]
