# Supplementary figures and images for: Mental health trajectories and Peer Refugee Helper engagement, among Afghan, Iranian and Syrian refugees and asylum seekers in Greece
Source: Glob Ment Health (Camb). 2025 Oct 13;12:e115. doi: 10.1017/gmh.2025.10068 (PMC12571684; doi:10.1017/gmh.2025.10068)

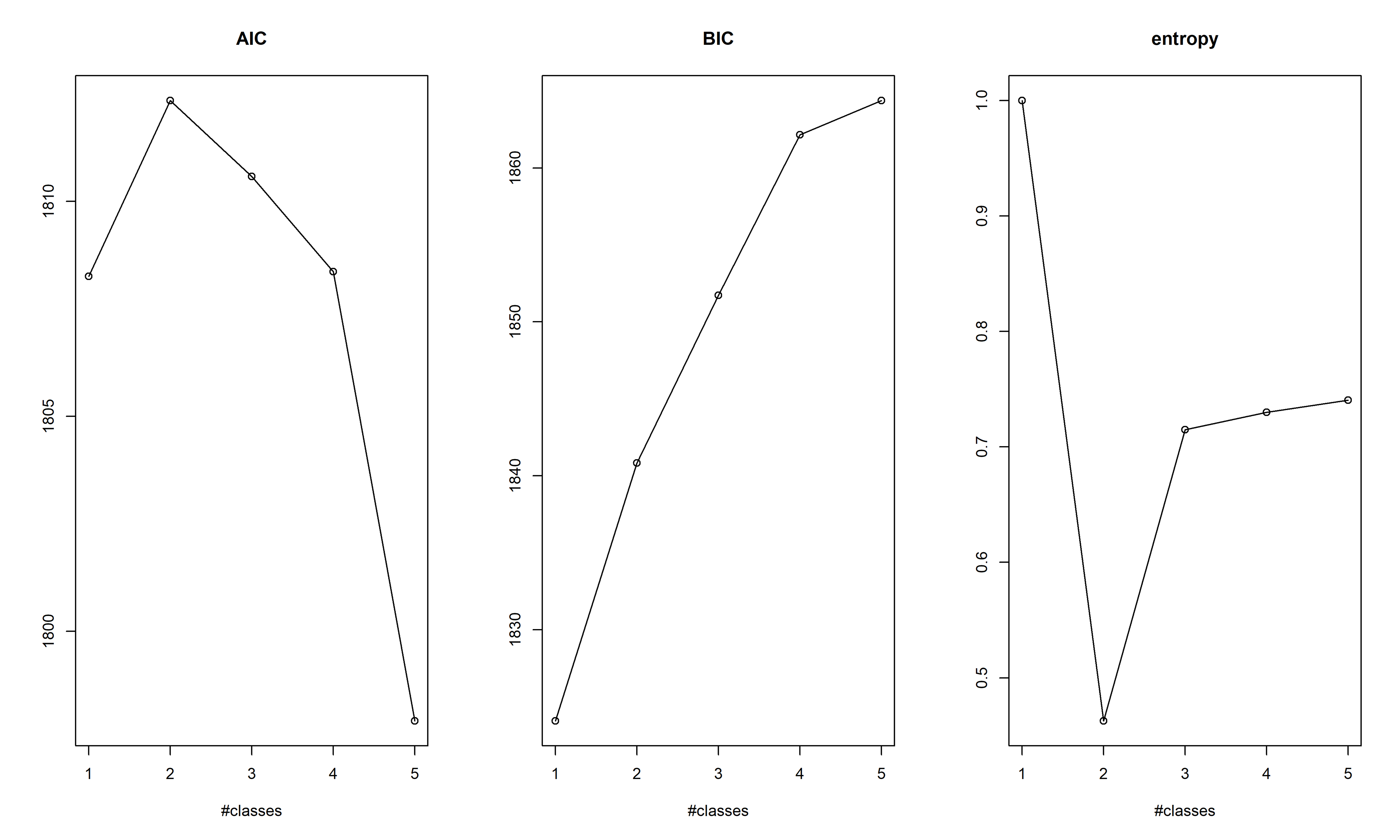

Supplement: Lavdas et al. supplementary material [file S205442512510068Xsup001.zip › S205442512510068Xsup002.tif]

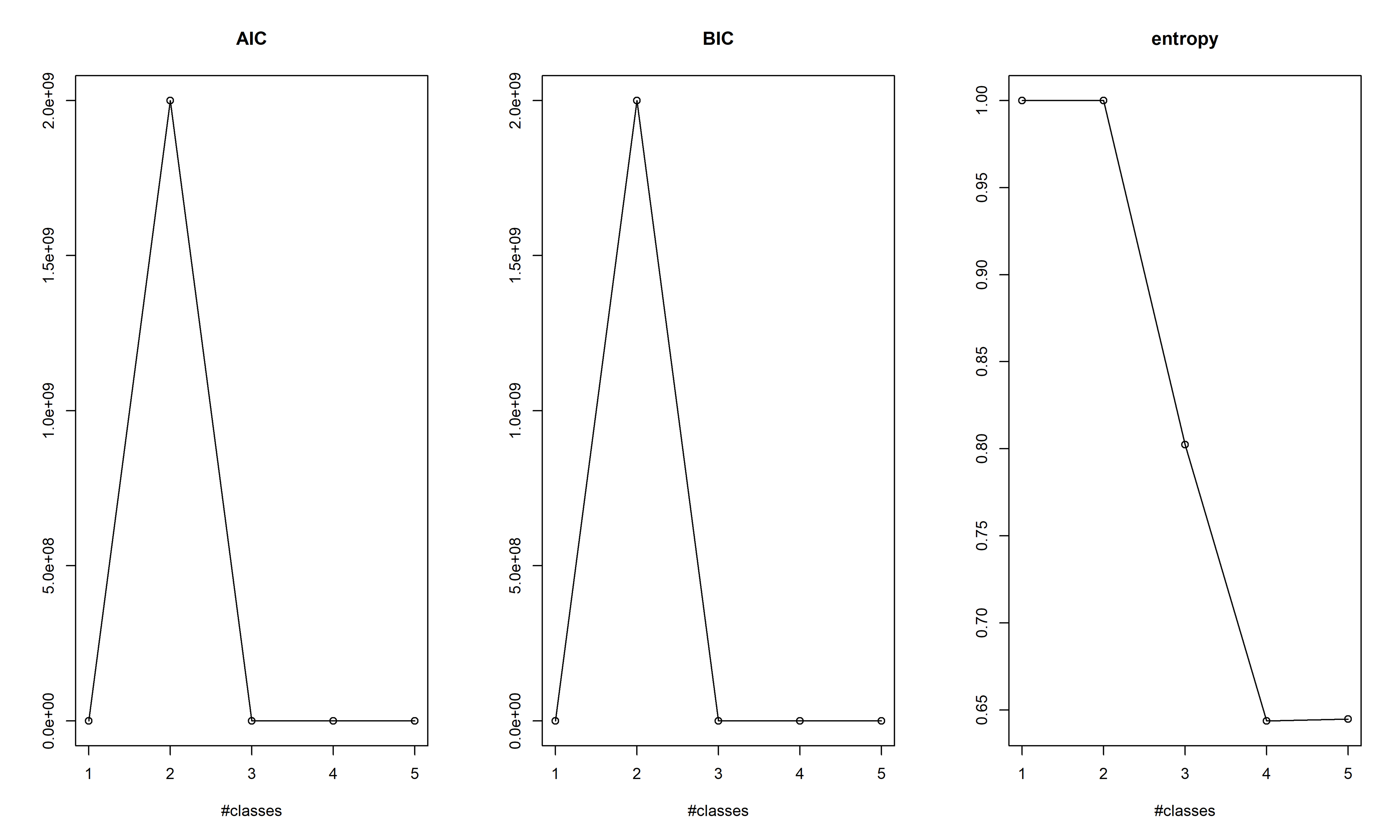

Supplement: Lavdas et al. supplementary material [file S205442512510068Xsup001.zip › S205442512510068Xsup003.tif]

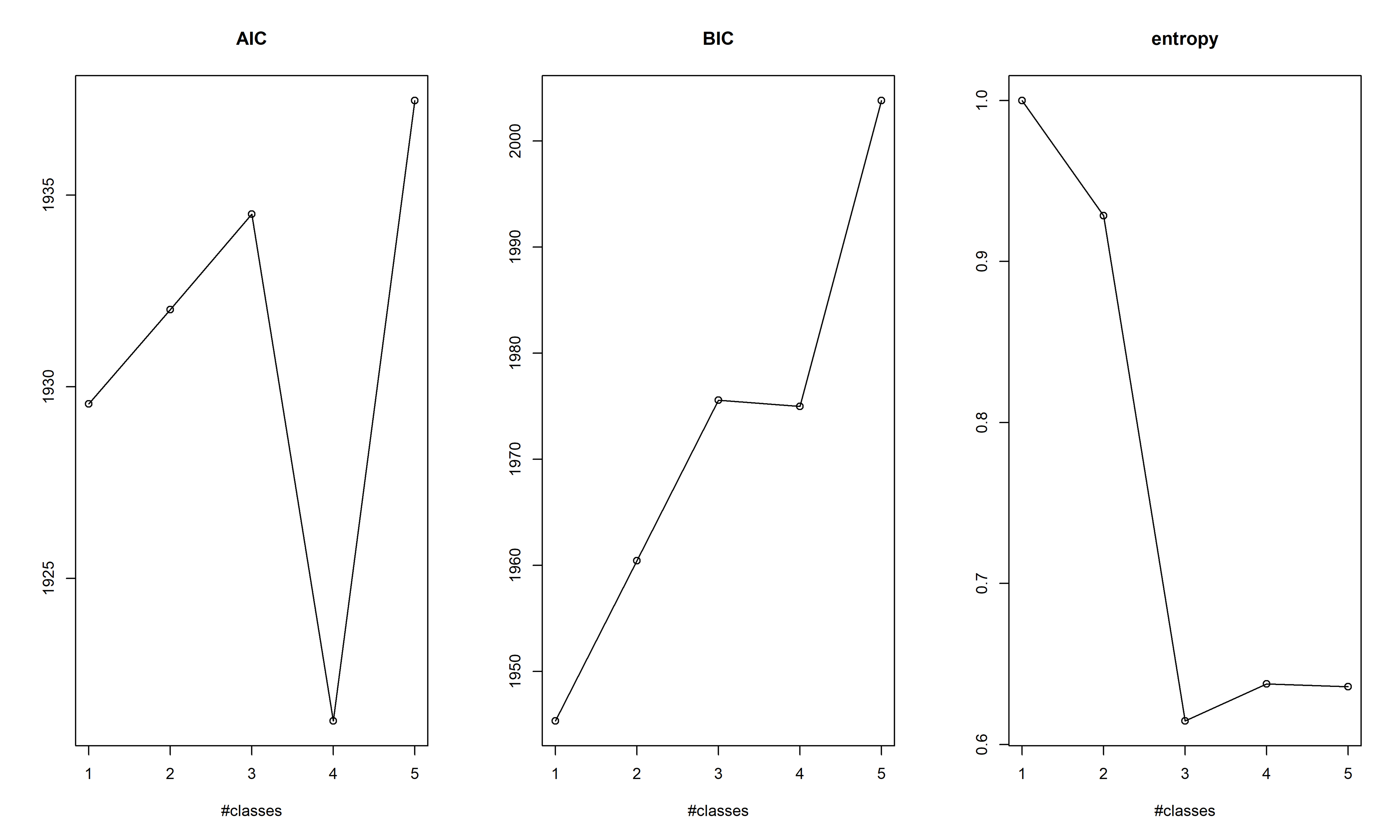

Supplement: Lavdas et al. supplementary material [file S205442512510068Xsup001.zip › S205442512510068Xsup004.tif]

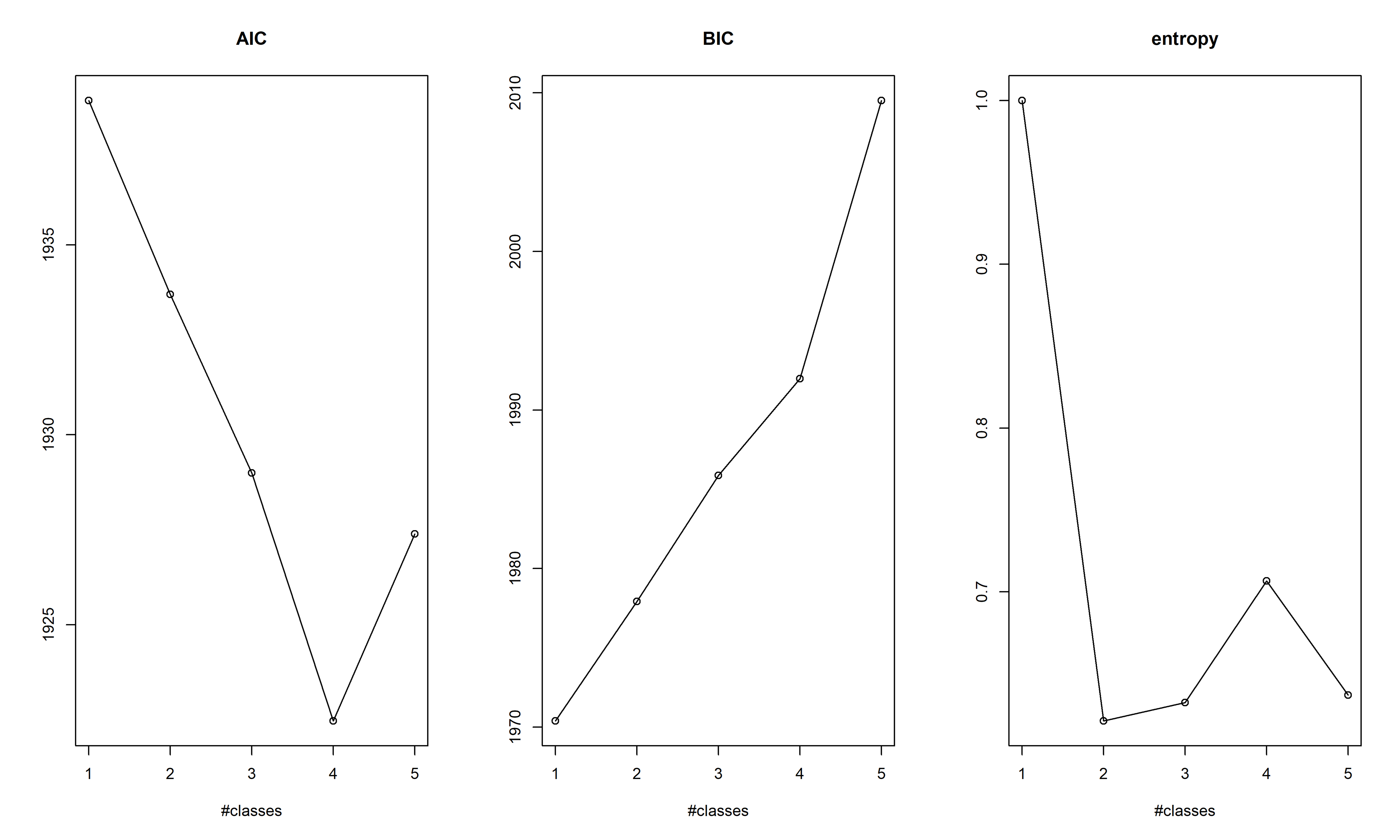

Supplement: Lavdas et al. supplementary material [file S205442512510068Xsup001.zip › S205442512510068Xsup005.tif]

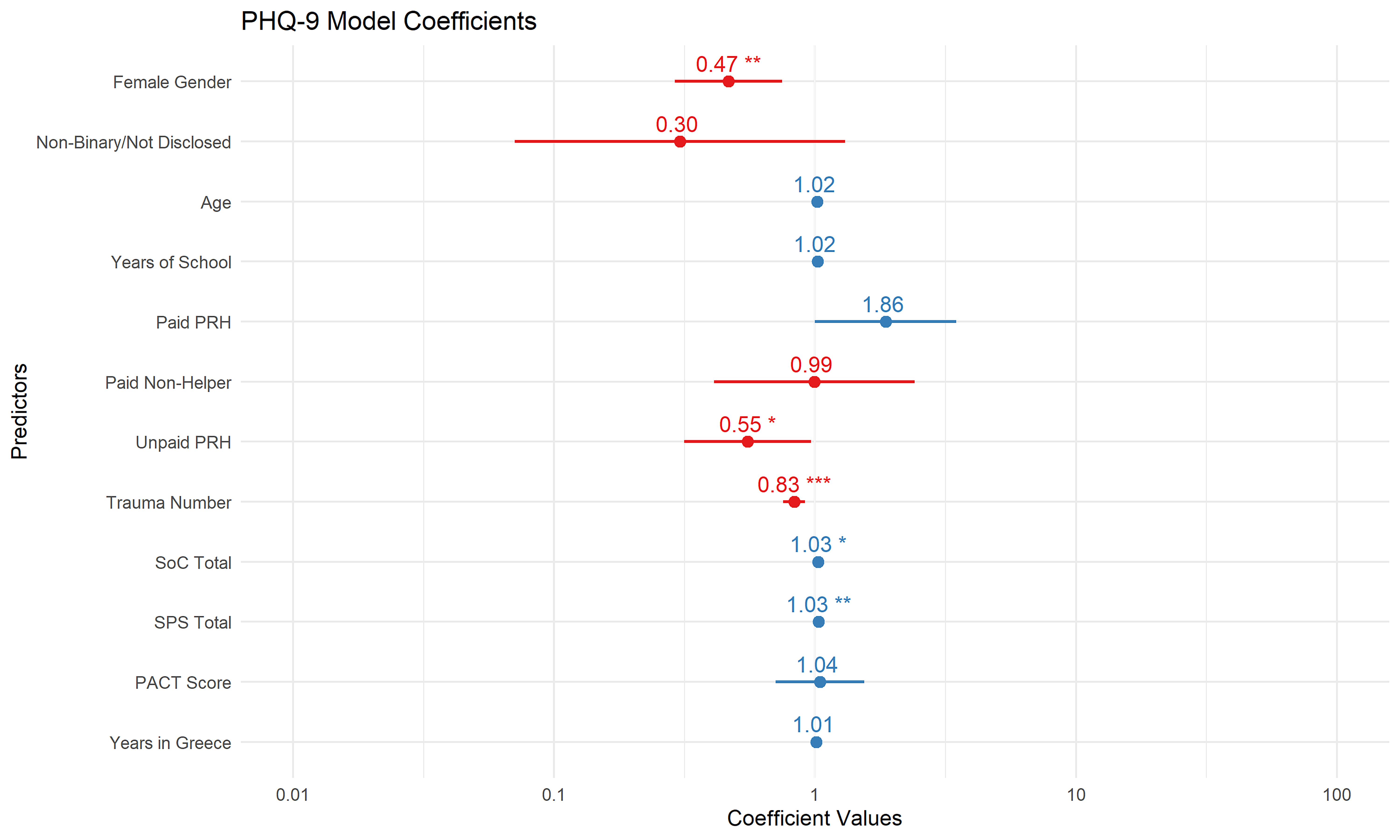

Supplement: Lavdas et al. supplementary material [file S205442512510068Xsup001.zip › S205442512510068Xsup006.tif]

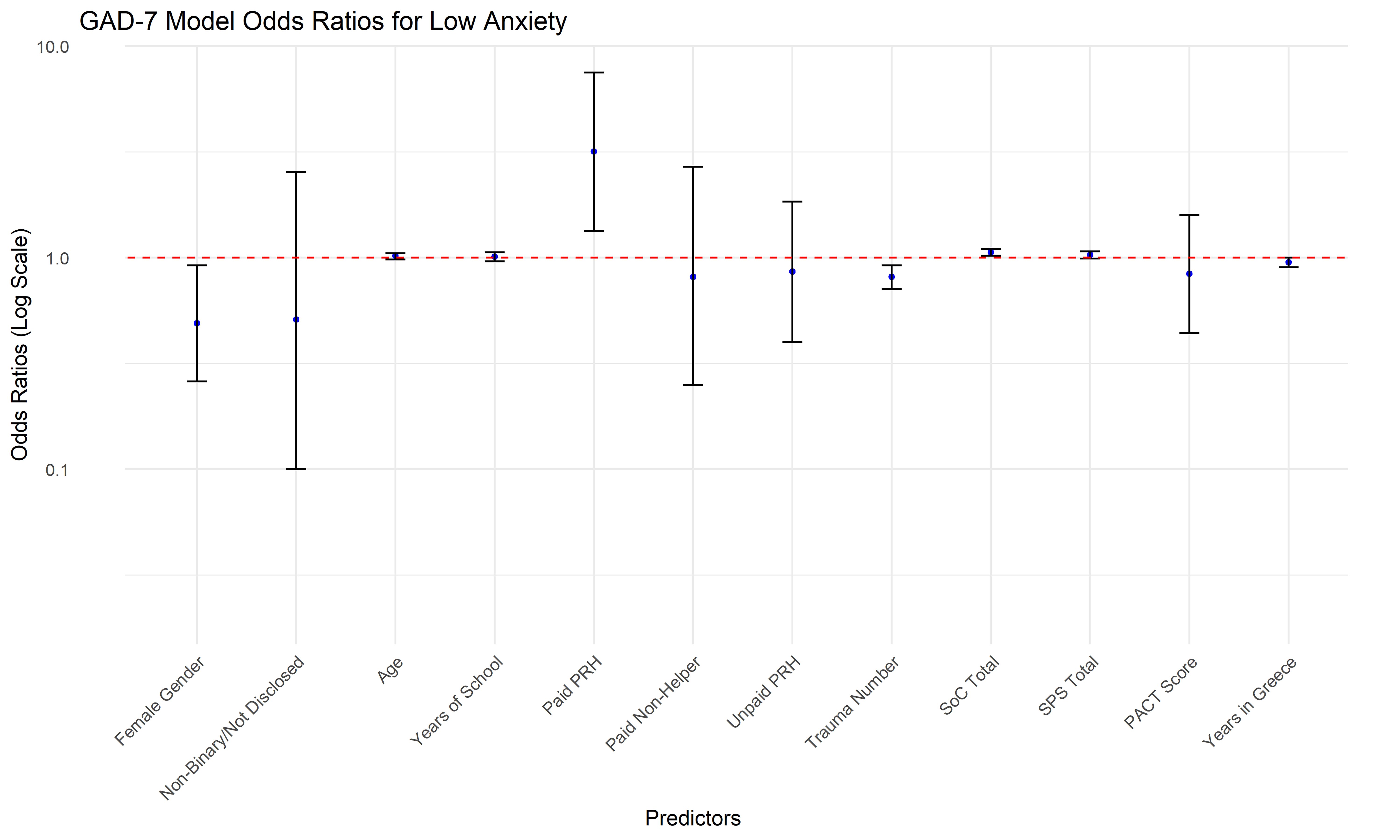

Supplement: Lavdas et al. supplementary material [file S205442512510068Xsup001.zip › S205442512510068Xsup007.tif]

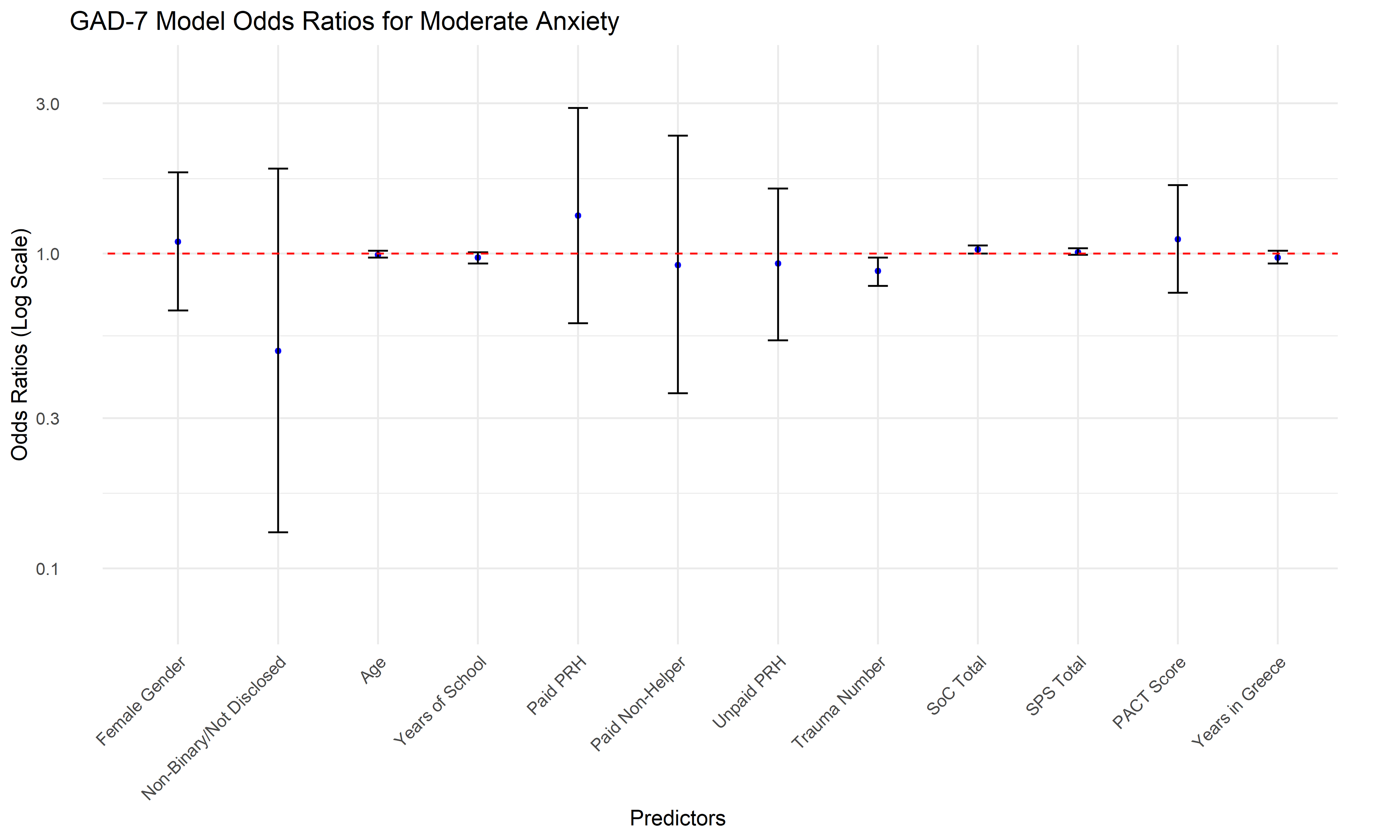

Supplement: Lavdas et al. supplementary material [file S205442512510068Xsup001.zip › S205442512510068Xsup008.tif]
